# Sequencing and analysis of the gene-rich space of cowpea

**DOI:** 10.1186/1471-2164-9-103

**Published:** 2008-02-27

**Authors:** Michael P Timko, Paul J Rushton, Thomas W Laudeman, Marta T Bokowiec, Edmond Chipumuro, Foo Cheung, Christopher D Town, Xianfeng Chen

**Affiliations:** 1 Department of Biology, University of Virginia, Charlottesville, Virginia, 22903, USA; 2 Academic Computing Health Science, Information Technology & Communication, University of Virginia, Charlottesville, Virginia, 22908, USA; 3 Institute for Genomic Research, Rockville, Maryland, 20850, USA; 4 Department of Microbiology, University of Virginia Health System, Charlottesville, Virginia, 22908, USA

## Abstract

**Background:**

Cowpea, *Vigna unguiculata *(L.) Walp., is one of the most important food and forage legumes in the semi-arid tropics because of its drought tolerance and ability to grow on poor quality soils. Approximately 80% of cowpea production takes place in the dry savannahs of tropical West and Central Africa, mostly by poor subsistence farmers. Despite its economic and social importance in the developing world, cowpea remains to a large extent an underexploited crop. Among the major goals of cowpea breeding and improvement programs is the stacking of desirable agronomic traits, such as disease and pest resistance and response to abiotic stresses. Implementation of marker-assisted selection and breeding programs is severely limited by a paucity of trait-linked markers and a general lack of information on gene structure and organization. With a nuclear genome size estimated at ~620 Mb, the cowpea genome is an ideal target for reduced representation sequencing.

**Results:**

We report here the sequencing and analysis of the gene-rich, hypomethylated portion of the cowpea genome selectively cloned by methylation filtration (MF) technology. Over 250,000 gene-space sequence reads (GSRs) with an average length of 610 bp were generated, yielding ~160 Mb of sequence information. The GSRs were assembled, annotated by BLAST homology searches of four public protein annotation databases and four plant proteomes (*A. thaliana*, *M. truncatula, O. sativa*, and *P. trichocarpa*), and analyzed using various domain and gene modeling tools. A total of 41,260 GSR assemblies and singletons were annotated, of which 19,786 have unique GenBank accession numbers. Within the GSR dataset, 29% of the sequences were annotated using the Arabidopsis Gene Ontology (GO) with the largest categories of assigned function being catalytic activity and metabolic processes, groups that include the majority of cellular enzymes and components of amino acid, carbohydrate and lipid metabolism. A total of 5,888 GSRs had homology to genes encoding transcription factors (TFs) and transcription associated factors (TAFs) representing about 5% of the total annotated sequences in the dataset. Sixty-two (62) of the 64 well-characterized plant transcription factor (TF) gene families are represented in the cowpea GSRs, and these families are of similar size and phylogenetic organization to those characterized in other plants. The cowpea GSRs also provides a rich source of genes involved in photoperiodic control, symbiosis, and defense-related responses. Comparisons to available databases revealed that about 74% of cowpea ESTs and 70% of all legume ESTs were represented in the GSR dataset. As approximately 12% of all GSRs contain an identifiable simple-sequence repeat, the dataset is a powerful resource for the design of microsatellite markers.

**Conclusion:**

The availability of extensive publicly available genomic data for cowpea, a non-model legume with significant importance in the developing world, represents a significant step forward in legume research. Not only does the gene space sequence enable the detailed analysis of gene structure, gene family organization and phylogenetic relationships within cowpea, but it also facilitates the characterization of syntenic relationships with other cultivated and model legumes, and will contribute to determining patterns of chromosomal evolution in the Leguminosae. The micro and macrosyntenic relationships detected between cowpea and other cultivated and model legumes should simplify the identification of informative markers for marker-assisted trait selection and map-based gene isolation necessary for cowpea improvement.

## Background

Cowpea, *Vigna unguiculata *L. Walp., is both one of the most important food and forage legumes in the semi-arid tropics and a valuable and dependable commodity for farmers and grain traders [1,2]. Of the ~21 million acres grown worldwide, 80% of cowpea production takes place in the dry savannah of tropical West and Central Africa, mostly by poor subsistence farmers in developing countries [2,3]. Despite its economic and social importance in the developing world, cowpea has received relatively little attention from a research standpoint and remains to a large extent an underexploited crop. Among the major goals of cowpea breeding and improvement programs is the stacking of desirable agronomic traits, such as those governing abiotic stress (drought, salinity, and heat) tolerance, photoperiod sensitivity, plant growth type, and seed quality with resistances to the numerous bacterial, fungal, and viral diseases and insect, invertebrate (nematode), and herbivorous pests [1,2]. Implementation of marker assisted selection and breeding programs is severely limited by a paucity of trait-linked markers and a general lack of information on gene structure and organization. Thus, relatively large genetic gains can likely be made with only modest investments in both applied plant breeding and molecular genetics.

The Leguminosae (Fabaceae) family consists of 757 genera and over 20,000 species [4]. Diversification commenced soon after the first identifiable legumes appeared in the fossil record ~56 million years ago (Mya), and all living legumes are thought to share a common ancestor that existed an estimated ~59 Mya [5]. The family is divided into several major clades [6], with most of the major crop and model species concentrated in the Papilionoideae clades "Halogalegina" (adapted to temperate climates, such as *Lotus *and *Medicago*) and "phaseoloid/millettioids" (warm-season species such as *Glycine *and *Phaseolus*). Cowpea belongs to the latter clade, along with the other major warm-season crops: common bean (*P. vulgaris*), pigeon pea (*Cajanus cajan*), and soybean (*G. max*) [7]. The split between *Glycine *and *Medicago *is dated at ~54 Mya and that between *Glycine *and *Phaseolus *at ~19 Mya [5,8].

The most significant progress in legume genomics has been made for the small genome model species, *M. truncatula *and *L. japonicus*, and for soybean (*G. max*), economically the most important legume crop species [8-13]. Large Expressed Sequence Tag (EST) collections are available for all three of these species [14], along with near complete genome sequences and well-developed genetic and physical maps [12]. The availability of genomics level information lags substantially in other legumes, although some progress has been made in pea (*P. sativum*), common bean (*P. vulgaris*), alfalfa (*M. sativa*), and peanut (*Arachis hypogea*) [7,15-18].

Little attention has been paid to gene characterization and the development of resources in cowpea [2,19] despite the fact that its genome size of 620 Mb is one of the smallest among the legumes and is at the lower end of plant genomes in general [20-22]. At the time of writing, fewer than 1,000 cowpea ESTs have been deposited in public databases [14] and most of the genomic DNA sequence available relates to either rRNA coding and spacer regions or represents anonymous sequence exploited for RFLP mapping. Increasing our knowledge of the structure and composition of the cowpea genome will help in the interpretation of genome evolution in this phaseoloid/millettoid clade and Papilionoideae in general, and will undoubtedly contribute substantially to efforts aimed at improvement of this crop.

While advances in high-throughput sequencing technologies make the prospects of whole genome sequencing possible, for most plant species the associated cost still remains prohibitive because of their genome size and complexity. Reduced-representation approaches, such as methylation filtration (MF) and C_o_t-based cloning and sequencing, have been developed to alleviate some of the difficulties presented by the presence of ubiquitous repetitive DNA [23-27]. Both techniques rely on gene enrichment for the recovery of genic sequences. While C_o_t-based selection separates low-copy from high copy sequence based on differential annealing [28,29], MF targets the hypomethylated fraction of the genome for cloning. The use of MF as an enrichment technique has been successfully demonstrated in maize, sorghum, soybean and tobacco [13,23,25,30-32]. Empirical comparisons suggest that the relative efficacy of MF and C_o_t-based enrichment the techniques is species dependent [25,26,33,34]. Here we show how MF was successfully applied in cowpea for the enrichment of gene-rich regions and report a detailed analysis of the resulting recovered sequences.

## Results and discussion

### Sequencing the gene-rich space of cowpea sampled by MF

In order to determine whether the application of methylation filtration (MF) technology could be used to enrich for hypomethylated gene-rich DNA from cowpea, a pilot study was carried out in which two whole genome shotgun libraries were generated from nuclear genomic DNA isolated from the cowpea variety UCR-1115. One library was made using the McrBC- strain of *E. coli *(the "unfiltered" (UF) library) and the other was made using the McrBC+ strain (the "methylation filtered" (MF) library). Nucleotide sequences were generated from one end of the inserted DNA in randomly selected clones picked from the UF and MF libraries, resulting in 1,152 and 864 sequence attempts, respectively (Table 1). The gene enrichment or filter power (FP) obtained was determined by comparison of the sequence reads from the UF and MF library clones to a highly-curated Arabidopsis protein database from which transposons and other repetitive elements had been removed [35]. Curation is an important step because it allows for the accurate determination of relative gene densities of the MF and UF sequence sets. Match rates were calculated over a range of BLAST e values (1e-5 to 1e-20) for the various MF and UF sequence reads and the resulting FP achieved by MF was 3.9 to 4.3, with a median value of 4.1 [see Additional file 1]. Given an estimated genome size of 620 Mb, a 4.1-fold enrichment predicts a hypomethylated, gene-rich space for cowpea of 151 Mb, about the size of the Arabidopsis genome. The enrichment following MF observed in cowpea was similar to the 3.2-fold enrichment observed in soybean (18) and better than the 2.4-fold enrichment found in *Phaseolus *(A. Budiman and J. Bedell, personal communication).

**Table 1:** Analysis of cowpea genespace sequence reads (GSRs) from methylation-filtered (MF) and unfiltered (UF) genomic libraries.

	MF Clones	UF Clones
Sequence Attempts	864	1152
Successful	820 (95%)	987 (86%)
		
Sequence Composition	Number of Clones (%)	Number of Clones (%)
		
Nuclear Inserts^a^		
Known	501 (61.1%)	236 (23.9%)
Unknown	216 (26.3%)	491 (49.7%)
Repetitive Elements	35 (4.3%)	200 (20.3%)
		
Cytoplasmic Inserts		
Chloroplast	23 (2.8%)	12 (1.2%)
Mitochondria	5 (0.6%)	1 (0.1%)
		
Extracellular Inserts		
Fungal	28 (3.4%)	16 (1.6%)
Bacterial	12 (1.5%)	31 (3.1%)
Viral	0 (0.0%)	0 (0.0%)

^a^Sequences from MF and UF libraries were compared to a highly-curated Arabidopsis protein sequence database as described in [35].

Based on empirically derived results from the Orion Sorghum GeneThresher™ project and a simulation conducted on finished Arabidopsis sequence [35], we estimated that in order to sequence tag some portion of ~95% of the genes in the cowpea genome, it would be necessary to generate ~252,000 MF sequence reads of an average read length of ~600 nucleotides [36]. This number depends heavily on the accuracy of the genome size estimate, and the probability that all genes lie within the sampled hypomethylated gene space. In the scaled-up version of the project, MF libraries were prepared from DNA isolated from the African cowpea cultivar IT97K-499-35 and 150,336 random clones were sequenced (147,744 inserts in both the forward and reverse direction, 2112 inserts in the forward direction only, and 480 inserts in the reverse direction only) (Table 2). In all, 298,848 MF gene-space sequence reads (GSRs) were generated, of which 263,425 or 88.1% were classified as successful sequencing attempts (i.e., were greater than 100 bp in length and at the time of their initial annotation were believed not to be derived from vector, microbial, fungal (yeast), viral or animal genomic DNA). Chloroplast (9985), mitochondrial (856), and transponson/retrotransposon-like (24,582) DNA sequences identified by BLAST (= 1e-10) were also removed. In total, the GSR dataset provided 160,696,129 nucleotides of raw sequence with the average length of a successful GSR being 610 nucleotides [see Additional file 2]. All GSRs, including organellar genome-derived sequences, as well as information on individual GSR lengths and other statistical analyses are available for interested individuals [36,37].

**Table 2:** Statistical data on cowpea genespace sequence reads (GSRs) and assemblies.

Total number of sequence attempts	298,848
Total number of successful sequences^a^	263,425
Success rate	88.1%
Usable read length (nucleotides)	
Minimum	100.0
Maximum	804.0
Mean	609.7
Median	644.0
Total Usable Bases	160,696,129
Number of Clusters	49,162
Number of GSR assemblies (contigs)	52,149
Number of singletons	70,679
Minimum Assembly Length (nucleotides)	64
Maximum Assembly Length (nucleotides)	23,920
Mean Assembly Length (nucleotides)	1,242
Median Assembly Length (nucleotides)	1,047
Total length of DNA represented by GSR assemblies and singletons	78,706,947
Number of GSR assemblies annotated by BLAST	23,372
Number of GSR singletons annotated by BLAST	19,881

^a ^Successful reads were greater than 100 bp in length and did not match DNA of extracellular (fungal, insect, bacterial or viral) and organellar (mitochondrion and chloroplast) origin when compared to available databases using blastx.

The 263,425 successful GSRs were clustered and assembled [38] into 52,149 GSR assemblies (contigs) and 70,679 singletons (Table 2). Relatively few singletons were generated by the assembly process indicating that the clustering of sequences was effective. The largest cluster (CL1) contains 1557 contigs with 17,407 members and the smallest cluster contains two GSRs. Clusters 2 through 30 contain from 354 to 57 component GSRs. Following assembly, the GSR dataset represents a nuclear coverage of ~78 Mb, which is equivalent to 52% of the sampled gene space and 12.7% of the total cowpea genome.

### Gene annotation and gene ontology analysis

To assess the efficiency of gene discovery following MF, the FASTA formatted cowpea GSRs were annotated and analyzed by both BLAST and Hidden Markov Model (HMM)-based algorithms [36]. The percentage of GSRs containing BLAST-identifiable gene sequences present in four public protein sequence databases and the proteomes of four completely or nearly completely sequenced plant genomes (Arabidopsi*s*, *M. truncatula*, rice, and poplar) was determined. As shown in Table 3, between 13% and 31% of the cowpea GSRs were annotated via the homology-based approach. Combining all of the distinct annotated GSRs, approximately 36% (95,364/263,425) of total cowpea GSRs could be assigned a putative function.

**Table 3:** Statistics on homology-based annotation of cowpea GSRs.

Annotation Databases	Number of Cowpea GSR Annotated	Distinct Accession Numbers	Annotation Database Size	Percent Matched Sequences
NCBI GenePeptide	78,787	23,561	3,440,254	29.91
UniProtKB PIR	67,807	12,921	283,416	25.74
UniprotKB Swiss-Prot	34,738	6,676	211,104	13.19
UniProtKB-TrEMBL	78,102	23,031	2,638,494	29.65
*Arabidopsis thaliana*	77,591	14,561	25,920	29.46
*Oryza sativa*	69,993	15,708	62,826	26.57
*Medicago truncatula*	61,711	7, 406	24,420	23.43
*Populus trichocarpa*	82,957	19,868	45,555	31.49

To determine the number of unique gene sequences represented in our dataset, BLAST comparisons to the NCBI GenBank peptide database [39] performed using the 52,149 GRS assemblies and 70,679 singletons resulted in the 41,260 annotated sequences. Of these annotated sequences, 19,786 had distinct GenBank accession numbers. By comparison, 23,561 distinct GenBank accession numbers were found by BLAST annotation of the 95,364 GSRs prior to assembly. Thus, the assembly process did not enhance or significantly compromise the identification of putative gene coding sequences.

The goal of reduced representation sequencing strategies such as MF is to capture as much gene complexity as possible without the laborious task of complete genome sequencing. Most plant genomes are thought to encode between 35,000 and 40,000 genes [22]. In legumes, gene density is estimated to be 1 gene per 6–10 kb [12,17,40-42]. With a gene space coverage of ~78 Mb captured by MF, the estimated minimum number of genes potentially tagged in our MF dataset should be between 7,800 and 13,000. In contrast to these predicted values, our annotation data clearly indicate that we tagged ~40,000 gene coding regions, representing a minimum of 19,786 distinct GenBank accession numbers. This latter number is also likely an underestimate, since we only included the single lowest e-value score per sequence. Some of the sequences matched multiple GenBank accession numbers and the second or third ranked e-values could represent additional coding regions on the same fragment.

**Figure 1:**
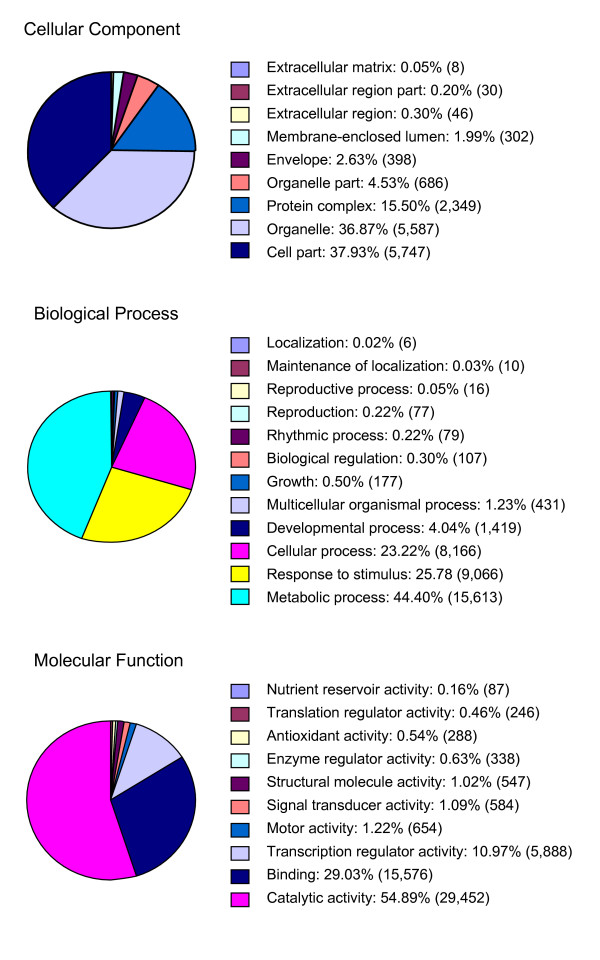
**Distribution of molecular function assignment for cowpea GSRs by GO annotation**. Gene Ontology (GO) annotations of cowpea GSRs were generated by Arabidopsis refseq BLAST searches and GSRs were assigned molecular functions using the complex search function, level 3 in the tree. A total of 77,591 cowpea sequences were annotated. Shown next to each functional category is the percentage of GSRs in each named category, followed in parenthesis by the number of annotated GSRs in the group.

To determine whether there was any bias in the enrichment for genes using MF, we made putative functional assignments for the individual GSRs based upon the most significant match obtained from database searches against the Arabidopsis GO annotation categories. As shown in Figure 1, the putative annotations were grouped into three top-level ontologies: cellular component, biological process, and molecular function. Approximately 29% (77,591/263,425) of the cowpea GSRs could be annotated in this way. Among those sequences that could be assigned a functional classification, the largest categories were catalytic activity and metabolic processes, groups that include the majority of cellular enzymes and components of amino acid, carbohydrate and lipid metabolism. Cellular binding activities (e.g., receptors) and gene products involved in cellular response to stimuli are among the second group of gene products. Among the GSRs assigned molecular function by GO annotation, 5,888 GSRs (~11%) had homology to genes encoding transcription factors (TFs) and transcription associated factors (TAFs). This value is similar to what was found by direct annotation of the GSR assemblies, in which ~5% (1042/19,786) of the total annotated sequences have this putative function assignment.

### Comparisons to ESTs of cowpea and other legumes

To better estimate the quality of our gene discovery, we compared the cowpea GSRs to available consensus EST-derived unigenes from cowpea and other legumes, including species closely and more distantly related (Table 4). In our analysis, we used 16,954 unigenes assembled from ~42,000 ESTs derived from root and leaf/stem transcripts of four different cowpea cultivars grown under both drought-stressed and well-watered conditions (Sarah Hearne, personal communication). Approximately 73.7% of cowpea ESTs-derived unigenes were represented in the GSR dataset as defined by blastn homology. Since the cowpea ESTs and GSRs come from different cowpea cultivars, nucleotide sequences differences are expected and, therefore, we also performed comparisons at the protein level using tblastx. These comparisons indicated an ~90% match rate. While the total number of ESTs available from cowpea is admittedly low, the high hit rate is a strong indication that the GSRs provide a broad and robust coverage of genic sequence.

**Table 4:** Results of BLAST comparisons of cowpea GSRs against various legume EST-derived unigenes.

Species	Number of Unigenes	Number of Matches with Cowpea GSRs^a^	Percent Match
*Glycine max*	63,669	46,858	73.60
*Glycine soja*	9,062	7,193	79.38
*Medicago truncatula*	35,969	24,703	68.68
*Lotus japonicus*	28,456	17,247	60.61
*Phaseolus coccineus*	7,666	5,858	76.42
*Phaseolus vulgaris*	9,474	8,394	88.60
*Vigna unguiculata*	16,954	12,501	73.70
All legume unigenes at LIS	167,046	117,585	70.39
Mean			74.43

^a^Comparisons between cowpea GSRs and EST-derived unigenes were performed as describe in the Materials and Methods using tblastx, with the exception of comparison to the the cowpea EST-derived unigenes where blastn was employed.

BLAST comparisons (tblastx) of the consensus ESTs-derived unigenes from other legumes and the GSRs showed that 88.6% of the unigenes from *P. vulgaris *matched cowpea sequences. This is not surprising since common bean and cowpea and phylogenetically very close. Not surprisingly, ESTs-derived unigenes from the more distally related *M. truncatula *and *L. japonicus *only had match rates with cowpea of 68.68% and 60.61%, respectively. The mean percentage of match for cowpea GSRs and available legume ESTs-derived unigenes was ~70%.

### Mapping cowpea GSRs to *M. truncatula *pseudomolecules

**Figure 2:**
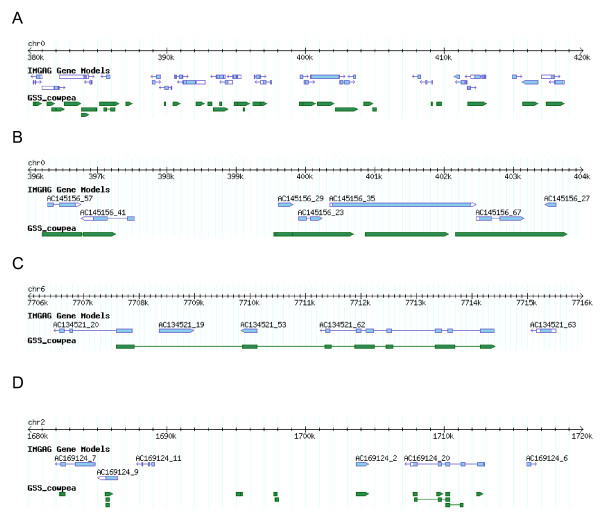
**Mapping of cowpea assemblies and singletons to the *M. truncatula *pseudomolecules**. GSR assemblies and singltons were mapped by tblastx searches to the *M. truncatula *chromosome-scale pseudomolecules available on the TIGR *M. truncatula *database. The broad green lines represent tblastx alignments; narrow lines connect High-scoring Segment Pairs (HSPs) derived from the same cowpea sequence. An HSP consists of two sequence fragments of arbitrary but equal length whose alignment is locally maximal and for which the alignment score meets or exceeds a threshold or cutoff score. A: An example of mapping cowpea contigs and singletons to a 40 kb region of chromosome 0 (which represents BACs that have not been anchored to the genetic map). B: A closer view of the same region from 396 k to 404 k. C: A region of *M. truncatula *chromosome 6 where a single cowpea GSR spans and has high quality tblastx matches to three distinct IMGAG gene models, indicating microsynteny. *M. truncatula *gene model AC134521_19 has no match in that region of the cowpea genome. D: A region of *M. truncatula *chromosome 2 where there are several GSR matches, but no *M. truncatula *gene model.

Using tblastx searches, 42,988 GSRs (24,075 GSR assemblies and 18,913 singltons) could be mapped to the *M. truncatula *chromosome-scale pseudomolecules available on the TIGR *M. truncatula *database [43]. The cowpea sequences are broadly distributed among the nine *M. truncatula *pseudomolecules [see Additional file 3]. Several examples of the mapping are shown in Figure 2. We were able to find over 500 cases where GSR assemblies/singletons map to at least 2 adjacent IMGAG genes along the pseudomolecules, indicating a significant level of microsynteny. We also found examples where along a syntenic region, there appears to be a gene missing in either cowpea or *M. truncatula *(Figure 2C). This could be due to either a gene insertion/deletion in one of the species. It is unlikely to be due to an annotation error in *M. truncatula*, since tblastx would detect sequence similarity in this region even if no gene model was predicted.

### Analysis of transcription factor (TFs) families

Plants devote ~7% of their genome coding capacity to proteins that regulate transcriptional activities [44-46]. Analysis of completed plant genome sequences suggests that are upwards of 60 TF families present in most plant genomes. In Arabidopsis [47,48] and *P. trichocarpa *[49,50] the 64 TF families vary in size from 1–2 members to over 100 members. Rice contains 63 of the dicot TF families [51,52], missing only the SAP1 family represented by only a single gene in both Arabidopsis and *P. trichocarpa*. About 43 of the known TF families and ~25 potentially novel plant TFs and TAFs were identified in an *in silico *analysis of the *M. truncatula *genome using the Medicago Gene Annotation Group (IMGAG) dataset as starting material [53]. Since ~5% of the cowpea GSRs showed some homology to known TF, we examined the distribution among the known TF families in vascular plants and in selected cases the complexity of cowpea TF families relative to what is found in other plant species.

BLAST homology searches were carried out using conserved domains for the 64 TF families previously defined in Arabidopsis [47,48] and tobacco [54]. One or more gene coding sequence for 62 of the 64 TF families previously identified in vascular plants could be identified in the cowpea GSR dataset, including sequences encoding the *SAP1 *TF family. Among the low copy TF families present in other plants, one member of each of the *LFY*, *NZZ*, and *ULT *families, two members of the *CCAAT-DR1 *and *Whirly *gene families, and 3 members of the *LUG *and *VOZ *gene families were present. Only the HRT-like and S1Fa TF families were not represented among the cowpea GSRs.

The ERF, WRKY, and *CONSTANS *(*CO*)/*CONSTANS*-like (*CO*-like) gene families were chosen for a more detailed analysis. These families are well characterized in other plant species and encode proteins that regulate a variety of plant developmental, stress, and growth responses. In Arabidopsis and *P. trichocarpa*, the ERF and WRKY families are among the largest TF families present, and the *CO*-like gene family has ~20 members. However, the most important criteria for selecting these TF families for analysis was that the gene products contain short, well-conserved DNA-binding domains that can be used to estimate diversity and phylogenetic relationships among family members, and to study gene family evolution [44,47,53,55]. Significant comparative information is available for the *CO*-like family in legumes [56].

### The ERF family

ERF transcription factors play important regulatory roles in plant responses to both biotic and abiotic stresses, sugar signaling, and determination of organ identity [57-59]. BLAST searches of the GSR dataset were based on a representative DNA binding domain from each of the ten major ERF subgroups (I-X) [58] using low stringency (cut off value = 10). The complete set of ERF domain-containing GSRs was assembled into contigs, each sequence was manually verified and false positives removed. This approach ensured that all possible gene family members, including the most highly divergent ones, were isolated. As a result, 111 ERF sequences were obtained, representing a minimum of 109 ERF genes. The minimum number is lower than the total number because a small number of sequences contain incomplete DNA-binding domains, and therefore some ERF sequences may represent the 5' and 3' ends of the same gene. The predicted minimum number of ERF gene family members present in cowpea (109) is similar to that predicted to be in the Arabidopsis genome (122–124) [44,58].

**Figure 3:**
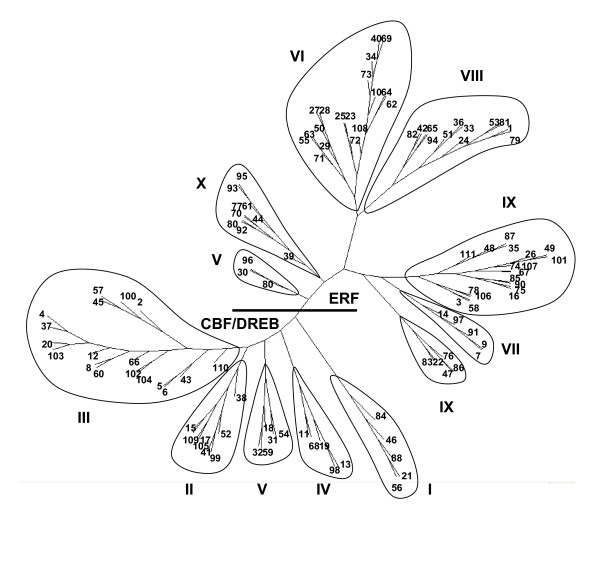
**The ERF gene family of cowpea transcription factors**. GSRs encoding the conserved DNA binding domain of ERFs were identified, the 111 cowpea ERF genes were arbitrarily assigned names, and the conserved domains were aligned using ClustalW. An unrooted phylogenetic tree was produced using the PHYLIP program based on the neighbor-joining method and presented using *PhyloDraw*. The cowpea ERF family is separated into two major clades. A line divides the CBF/DREB subfamily from the ERF subfamily. Subgroups, indicated by roman numerals, were identified as described in [58]. For additional information see Additional file 5 and Additional file 6.

The phylogenetic structure of the cowpea ERF gene family (Figure 3) is similar to that of Arabidopsis. Two major clades are recognized, the ERF and CBF/DREB subfamilies, consisting of the ten major subgroups. Subgroup IX contains the largest number of genes and is divided into two branches. The placement of Group V genes in cowpea differs significantly from what is observed in Arabidopsis [44], with the cowpea Group V ERFs forming separate branches in both the CBF/DREB and ERF clades. This may be a more common feature of the ERF family, since the Group V genes of tobacco also form separate branches in both the CBF/DREB and ERF clades [54], and in rice, some Group V genes cluster with Group XI [58].

The high degree of similarity in the phylogenetic arrangement of ERF genes between cowpea and Arabidopsis indicates that it should be possible to use such analyses to identify potential targets for cowpea improvement. We also constructed a phylogeny that contained the ERFs of cowpea and those of other plant species whose biological function has been reported (data not shown). Using this type of analysis we were able to identify the closest cowpea homologues of *CBF1*, *DREB1A*, *TINY*, *CaPBF1*, *ORCA3 *and *Pti4*, ERFs known to be regulators of important agronomical traits such as drought, salt tolerance, freezing tolerance, and disease and pest resistance [see Additional file 4].

### The WRKY family

The WRKY TFs regulate responses to biotic and abiotic stresses, senescence, germination, and a number of developmental processes [60-63]. Each WRKY transcription factor contains at least one conserved ~60 amino acids region (the WRKY domain) with the peptide sequence WRKYGQK at the N-terminus and a Zn-finger motif at its C-terminus [64]. The ancestral-type WRKY TF (Group I) contains two WRKY domains, one N-terminal and the other, C-terminal. All other genes contain just one WRKY domain and are classified into Groups IIa, IIb, IIc, IId, IIe and III on the basis of their primary amino acid sequence and structure of their Zn-finger motifs. A BLAST search of the cowpea GSRs was performed with each of the WRKY domains from the various subgroups and with both the N-terminal and C-terminal domains from Group I. A total of 79 contigs, containing at least part of a WRKY domain, were obtained. Discovery of WRKY genes was technically more difficult than the ERF genes because most WRKY domains are interrupted by an intron separating the WRKY and Zn-finger parts of the domain. The effect of this intron was that frequently only the 5'- or 3'-end of the WRKY domain was present in the assembled contigs. Nevertheless, it was possible to estimate the minimum number (e.g., if all 5'- and 3'-ends were joined) and maximum number (e.g., if all 5'- and 3'-ends were not joined) of WRKY genes present to be, 53 and 79, respectively. These values are consistent with the prediction of 72 WRKY genes in Arabidopsis [44].

**Figure 4:**
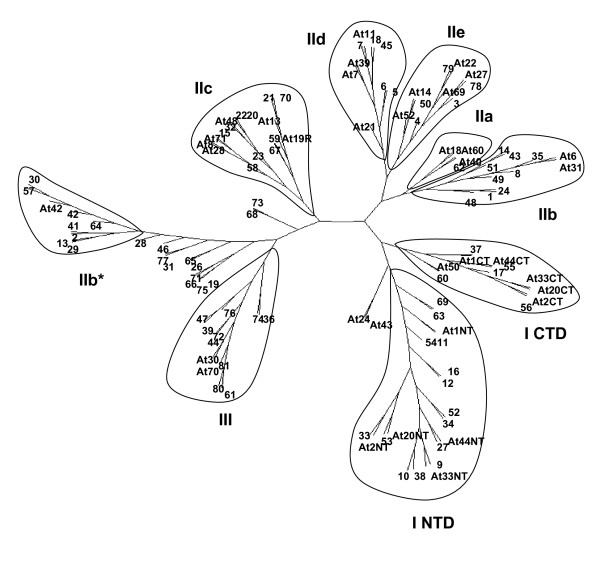
**The WRKY gene family of cowpea transcription factors**. GSRs encoding the conserved WRKY domains were identified, the 79 WRKY genes were arbitrarily assigned names, and the conserved domains were aligned using ClustalW. An unrooted phylogenetic tree was produced using the PHYLIP program based on the neighbor-joining method and presented using *PhyloDraw*. The comparison includes a small number of Arabidopsis WRKY TF genes representative of each group. Cowpea WRKY genes are indicated by the prefix Vu, and Arabidopsis genes by the prefix At followed by their number and group. Groups are indicated by roman numerals. Group I sequences include both the N- and C-terminal domains (I NTF and I CTD, respectively); subgroup IIb* is an artifact in the ClustalW sequence alignment caused by the truncated nature of some of the domains. For additional information see Additional file 7 and Additional file 8.

A phylogenetic tree of the cowpea WRKY TFs based on their WRKY domain sequence, and including some key Arabidopsis WRKY genes, was generated (Figure 4). This revealed that the organization of the gene family in cowpea is similar to that in Arabidopsis, with the exception of the Group IIb genes that appear to fall into two distinct clades. This is likely an artifact caused by the presence of some truncated WRKY domains in the analysis, because of all the genes found in the cluster designated IIb*, only two (*VuWRKY13 *and *VuWRKY29*) have full-length WRKY domains.

Cowpea homologs of functionally characterized WRKY genes could also be identified. These include *VuWRKY44 *and *VuWRKY36*, homologs of *AtWRKY70*, a TF that functions at the intersection of salicylic acid and jasmonic acid signaling during defense responses [65], *VuWRKY35*, a homolog of *AtWRKY6*, whose product plays a role in regulating senescence [66], and *VuWRKY27 *and *VuWRKY55*, homologs of TRANSPARENT TESTA GLABRA (*TTG2*/*AtWRKY44*), which plays a key role in trichome and seed coat development [67].

### *CONSTANS *and the *CONSTANS*-like gene family

The timing of flowering is an important agronomic trait in crop plants [68-71]. Many genes involved in photoperiod responsiveness are functionally conserved in monocot and dicot species [72,73]. As a result, it was possible for us to identify GSRs encoding many members of various gene families involved in light perception (e.g., *PHY*, *CRY*), as well as GSRs encoding components of the signal transduction pathways connecting photoperiod and phytohormonal stimuli in the induction of flowering. For example, the interaction between the products of the *CONSTANS *(*CO*) and *FLOWERING LOCUS T *(*FT*) genes underlie long- and short-day responsiveness [70-74]. *CO *encodes a TF that plays a central role linking the circadian clock to genes controlling meristem identity [73,75]. *CO *and members of the *CO*-like gene family are defined by the presence of two conserved domains, a Zn-finger domain that resembles a B-box domain near the N-terminus and a CCT domain [75] near the C-terminus [76]. In Arabidopsis, the *CO*-like gene family consists of three broad groups: Group I which includes *CO *and factors with two Zn-finger B-boxes near the N-terminus, Group II with one B-box, and Group III with one B-box and a second diverged Zn-finger. There is a surprising amount of disagreement in the literature about the number of CO-like genes present in Arabidopsis, with values ranging from 17 [76] to between 33 – 51 genes [44,77], due to variation among researchers in defining what constitutes a CO-like gene. Some researchers accept "CO-like genes" that only contain a CCT domain and but not a B-box. Since CCT domains are present in other TFs (e.g., ZIMs) and the B-box is a feature absolutely required for CO function [73,75], in our analysis we only considered those genes that fit the stricter definition (i.e., *CO *and *COL1-COL16*). As a result, 23 cowpea CO-like genes were identified.

**Figure 5:**
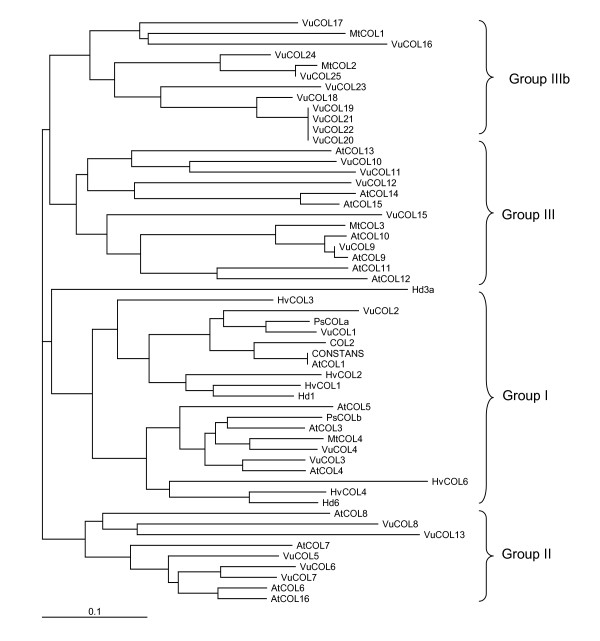
**The *CONSTANS *(*CO*) and *CO*-like gene family from cowpea**. GSRs encoding the conserved DNA binding domains of *CONSTANS *(*CO*) and *CO*-like TFs were identified and assembled into contigs, the putative genes were arbitrarily assigned names, and the B1 and/or B2 domains (depending on the gene) were manually excised and aligned using CLUSTALW. An unrooted phylogenetic tree was produced using the PHYLIP program based on the neighbor-joining method and presented using *PhyloDraw*. The comparison includes a small number of Arabidopsis, barley, pea, rice and *M. truncatula CO*-like genes. Cowpea genes are indicated by the prefix Vu; Arabidopsis genes by the prefix At; *M. truncatula *genes by the prefix Mt, pea by the prefix Ps, barley by the prefix Hv, and rice by the prefix Hd. The major groups are indicated by roman numerals. The bar indicates the percent sequence divergence. For additional information see Additional file 9.

The cowpea CO-like gene family has several interesting features. Two cowpea genes, *VuCOL1 *and *VuCOL2*, clustering together within Group I are clearly the closest homologs of the Arabidopsis *CO*, *COL1*, and *COL2 *genes (Figure 5). Their corresponding functional role is currently under investigation. The cowpea Group II and Group III CO-like genes are similar in number to those in Arabidopsis, whereas the Group IIIb, consisting of *VuCOL16 to VuCO25*, has no counterpart. Two *M. truncatula *homologs of cowpea Group IIIb genes exist suggesting that perhaps this branch is legume specific. Four CO-like genes are known in monocots (two each from rice and barley) that similarly contain only a single B-box domain [76]. The functional role of these Group IIIb CO-like genes in cowpea remains to be elucidated.

Vernalization acts to promote flowering by repressing the expression of another floral regulator, the MADS-domain protein termed FLOWERING LOCUS C (FLC) in Arabidopsis. Cereals appear to be missing FLC-like genes in their genomes, with the corresponding role being carried out by an unrelated Zn-finger TF [78]. FLC is also conspicuously absent in the legumes [53,56]. Consistent with this previous observation, we were unable to identify a FLC homolog in the MADS-domain TF family represented in the cowpea GSRs (J. Opoku, P. Rushton, and M.P. Timko, unpublished observations).

### Genes controlling symbiosis and biotic stress responses

Legumes form mutually beneficial symbiotic associations with arbuscular mycorrhizal (AM) fungi and bacteria collectively known as rhizobia that are of tremendous agricultural importance [79,80]. Establishing a fully functional symbiosis requires the successful completion of numerous steps, beginning with the recognition of chemical signals exchanged between the plant and bacterial/fungal symbiont and culminating in the differentiation of functional symbiotic cells/tissues. The process is the result of tightly regulated biochemical and molecular interactions between the legume host and its symbiont [81-86]. While the processes of nodulation and AM invasion have been extensively examined in other legume species, little experimental work has been done in cowpea. We searched the GSRs for homologs of genes known to be involved in nodulation and AM-legume symbiosis and identified the following: NFR1/NFR5, receptor kinases that perceive the bacterial derived signal in nodulation; SYMRK, receptor-like kinases that integrate perception of the signal and initiate symbiosis; NIN1 and members of the NIN-like family of TFs; GRAS-domain family proteins, such as NSP1 and NSP2; DMI1 and DMI3; putative plastidic ion channel protein components CASTOR and POLLUX; nucleoporin NUP133, required for the induction of Ca2+ spiking in nodule development; nodulin (NOD)-genes and genes encoding various nodule-specific proteins.

A large number of genes are involved in plant responses to biotic stresses (e.g. bacteria, fungi, insects, nematodes, and parasitic plants) [87]. Both resistance (R) genes and genes encoding components of the signaling pathways activated by the R genes in the defense response have been extensively studied [88-90]. The largest class of R genes encodes intracellular proteins containing a nucleotide binding-site (NBS) and C-terminal leucine-rich repeats (LRR). The NBS family can be divided into multiple subfamilies based upon the presence or absence of other domains, such as a *Toll*/interleukin receptor domain (TIR) region, a coiled-coil (CC) domain, and a BED finger and/or DUF 1544 domain [87,89]. Comparison of legume and non-legume resistance gene homologs indicates that legume genes possess a unique evolutionary history, with many clades either unique to legumes or expanded within legumes [91]. Preliminary homology-based analysis of the cowpea GSRs dataset using previously identified conserved NBS domains from cowpea R genes [92], and NBS and LLR domains from R genes of other legume and non-legume species, identified > 500 R genes and R gene candidates. A fuller analysis of the diversity and phylogenetic relationships of these R genes is now underway.

In addition to the R genes, many of the conserved signaling components of the disease resistance response pathways are present in the cowpea GSRs, including *NDR1*, *RPM1*, *COI1*, *EDS1*, *EDS5*, *PR5*, *SGT1*, *RPS5*, and *RIN4*. Consistent with previous reports in other plant species, expression of these genes in cowpea has been shown to be activated by treatment with salicylic acid, jasmonic acid, or ethylene, and by wounding or by attack by the parasitic angiosperm *Striga gesnerioides *[93,94].

### Categories and distribution of simple sequence repeats (SSRs)

Simple sequence repeats (SSRs) provide the basis of the most flexible and most widely exploited molecular marker systems for marker assisted selection and genetic analysis in crops [95,96]. A total of 30,877 GSRs (11.7% of the total) contain at least one SSR. Of these, 3,717 have been allocated a putative function through a BLAST-homology based search. The SSRs vary in both repeat composition and repeat length, with di- and tri-nucleotide motifs being the most prevalent. AT/TA repeats were the commonest dinucleotide motif. Among the trinucleotide repeats, AT-rich groups (AAT > ATA > TAT > ATT) predominated in the total GSR dataset, whereas ATA and TTC were the most frequently occurring trinucleotide repeats among the annotated GSRs. The nature and size distributions of the cowpea SSRs 24 bp or greater is summarized in Table 5 and additional information can be found at [37].

**Table 5:** Summary statistics from analysis of cowpea GSRs for the presence and type of simple sequence repeats (SSRs)

Motif ^a^	Number in Total GSR/Number in Annotated GSR	Minimum Copy Number	Mean Copy Number	Maximum Copy Number
Mono	176	25	33	123
	38	25	29	52
Di	10245	12	25	38
	749	12	23	212
Tri	1704	8	19	230
	258	8	13	49
Tetra	349	6	12	160
	75	6	10	26
Penta	340	5	12	99
	51	5	7	44
Hexa	718	4	7	60
	199	4	6	24
Hepta	425	3	8	69
	59	4	6	24
Octa	493	3	7	70
	36	3	4	10
Ennea	453	3	5	64
	73	3	4	12
Deka	537	2	5	24
	56	3	4	9
>Deka	17403	2	2	55
	2845	2	3	18

^a ^Repeat motif by nucleotide unit length of more than 24 bp repeat length

## Conclusion

Among the legumes, comparative genetic mapping established early on that linkage relationships were well conserved between closely related genera [97-102]. As more sequence information has become available, the extent to which both macro- and microsynteny relationships exist has emerged. Despite significant differences in genome size, a high level of macrosynteny exists between *Medicago *and the Galegoids (such as alfalfa, pea, chickpea, and *Lotus*), whereas less macrosynteny is observed between *Medicago *and the Phaseolids (such as soybean and mungbean) [103-110]. The present work provides a firm foundation for detailed comparative studies of cowpea with other warm season legumes, which apart from soybean are as yet poorly represented. Many of the cowpea coding regions were readily mapped to the *M. truncatula *pseudomolecules, allowing for future efforts aimed at the dissection and analysis of regions of macro- and microsynteny. Such information, in combination with improvements of the current cowpea genetic map [111] will facilitate positional cloning of key genes of agronomic interest.

The development of genomic scale information and its use to conduct global transcriptomic, proteomic, and metabolomic analyses is a major goal of the legume research community [16,22]. Such analyses are already well advanced in the model legumes [112-117]. The provision of extensive genomic data for cowpea, a non-model legume with significant importance in the developing world, represents a significant step forward in legume research. Not only does the gene space sequence provided here enable the detailed analysis of gene structure, gene family organization and phylogenetic relationships within cowpea, but it also facilitates the further characterization of syntenic relationships among cultivated and model legumes. Ultimately these types of studies will contribute to determining patterns of chromosomal evolution in the Leguminosae. The determination of micro- and macrosyntenic relationships between cowpea and other cultivated and model legumes should assist in the identification of informative markers for use in marker-assisted trait selection and map-based gene isolation. The GSRs sequences we have generated also provide a resource for future studies of gene expression within cowpea. The development of oligonucleotide-based microarrays for functional genomics analysis is currently underway in our laboratory and this resource should soon be available for the legume community. We hope that the information and materials provided here will stimulate the broader goal of the genetic improvement of cowpea, which is a priority for the alleviation of the burden of biotic and abiotic stresses on subsistence farmers in developing parts of the world.

## Methods

### Plant materials

Seeds of two cowpea cultivars were used in these studies. Cultivar UCR-1115 (obtained from Dr. Jeff Ehlers, Department of Botany and Plant Sciences, University of California, Riverside, CA) was used in the pilot study and IT97K-499-35 (obtained from Dr. Mohammad Ishiyaku, Ahmadu Bello University, Zaria, Nigeria) was used in the full scale gene space sequencing project. Seeds of both genotypes are publicly available on request.

### Genomic library construction and methylation filtering

Genomic DNA was purified from isolated nuclei of 1 month-old cowpea leaves [24] except that OptiPrep™ (Axis-Shield PoC, Oslo, Norway) was used [35]. Purified nuclear DNA was sheared using a Hydroshear apparatus (GeneMachines, San Carlos, CA, USA) and the sheared fragments end-repaired using End it™ kit (Epicentre, Madison, WI, USA). *Bst*XI adaptors (Invitrogen, Carslbad, CA, USA) were ligated to the end-repaired fragments, and the ligation products were size separated by agarose gel elecrophoresis. DNA fragments ranging from 0.7 – 1.5 kb were extracted from the gel and ligated to dephosphorylated, *Bst*XI-digested pOT2 vector (from the Berkeley Drosophila Genome Project, BDGP) for use in library construction. Ligation reactions were transformed into McrBC+ and McrBC- strains of *Escherichia coli *for generation of methylation filtered (MF, GeneThresher^® ^technology) and unfiltered (UF) libraries, respectively. The MF and UF libraries were plated onto selective agar medium, recombinant colonies were randomly picked using a Genetix Q-bot robot (Research Genetics, Carlsbad, CA, USA) and the selected clones arrayed individually into glycerol storage medium in 384-well microtiter plates for archiving and storage at -80 C.

In a Pilot Study, clones were picked at random from the MF and UF libraries, plasmid DNA isolated from each clone, and sequenced from one end using an ABI 3730 (PE Applied Biosystems, Foster City, CA). The resulting sequence data was analyzed as described below to estimate gene enrichment or filtering power. The purity of the nuclear genomic DNA preparation was determined by measuring two sources of DNA contamination: extracellular DNA (e.g., fungal, insect, bacterial or viral) and organellar DNA (i.e., mitochondria and chloroplast) [35].

Library preparation and clone picking for the full-scale genespace sequencing project was carried out as in the Pilot Study. The quality and filtering capacity of each library made from IT97K-499-35 was determined. A total of 150,336 randomly selected recombinant clones were picked from the MF libraries using a Genetix Q-bot robot (Research Genetics, Carlsbad, CA, USA), arrayed and stored individually in 384-well microtiter plates. One sequence attempt was made from each end of the insert fragment for each of the individual clones. A successful sequence read met the following criteria: at least 100 contiguous bases of good quality, insert sequence following vector and quality trimming performed using the -trim_alt option of the Phred basecaller software program [118] and was not derived from organellar (chloroplast and mitochondrion), vector, transposon/retrotransposon, microbial, fungal (yeast), viral or animal genomic DNA as determined by BLAST searches of relevant public databases. A significant similarity score equal to or less than 1e-10 was used.

### Bioinformatic analyses

The gene enrichment or filter power (FP) was calculated by comparing the rate of gene discovery between MF and UF sequences and is based on the proportion of matches of MF sequences compared UF sequences over a range of e-values from 10e-5 to 10e-20, such that all matches better than the given e-value are tabulated [see Additional file 1]. To ensure high quality, unique sampling events, reads were chosen that contained at least 100 contiguous Phred Q20 bases and only one read per clone was used. Detection of genes was accomplished by a blastx search (parameters: -e 0.01; -b 5; -v 5) of the curated Arabidopsis protein database. Aside from the curation of the Arabidopsis database to remove repetitive elements, matches to proteins annotated as hypothetical were not counted as hypothetical genes are often false gene predictions or unknown repetitive elements. The Arabidopsis protein set, which was used for the FP calculations and assessment of cross-genome annotation potential, is described elsewhere [35].

Raw sequence reads and vector-trimmed gene-space sequence reads (GSRs) are stored in FASTA format on a publicly available PostgrelSQL relational database [36]. The primary sequence dataset consists of 263,425 FASTA formatted cowpea GSRs with an average length of 610 bp (see Additional file 2). Sequence annotation and analysis was performed using both BLAST and Hidden Markov Model (HMM) based algorithms [36]. For homology-based annotation, each GSR was searched with blastx, with cutoff expectation (e) value of 1e-8, against the UniProtKB-TrEMBL Database [119], UniprotKB-Swiss-Prot Database [120], NCBI GenBank Proteins Database [121], and UniProtKB-PIR (Protein Information Resource) Database [122] and the Arabidopsis, rice, *Medicago*, and poplar protein datasets as described in [36].

Sequence assembly (clustering) was done with the TGI clustering tool (TGICL) from Harvard University [38] which uses megablast to group overlapping sequences into clusters, then assembles the clusters using CAP3 [123]. Parameters used were a minimum overlap length of 30 bp with 94% sequence identity.

### Analysis of gene ontology

Gene Ontology (GO) annotations of GSRs were generated by Arabidopsis refseq BLAST searches. Accession numbers from the BLAST annotation were used to look up GO term and name for each annotatable sequence. For each cowpea GSR that had a GO term, we tracked backwards by the shortest path to an ancestor at the third level. We used the GO MySQL database file go_20070204-seqdblite-tables.tar.gz [124]. Although geneontology.org distributes the GO SQL database in MySQL we prefer PostgreSQL. Only the usual minor conversions were necessary to load the GO data into PostgreSQL. The GO database includes a number of related tables. SQL joins allow accession numbers to be associated with GO terms. For the sake of efficiency (to avoid repeating a SQL join on four tables) we created a new table assoc_term_seq to capture the available associations between table's term, association, gene_product, and seq. One additional query gave us the term, and a second query gave us the closest level 3 ancestor for that term. A Perl script handled the database connections, the gathering of the cowpea refseq accession numbers, iteration of the SQL queries over the cowpea accession numbers, and the accumulation of counts into the GO category terms.

### EST comparisons and mapping of GSRs to *M. truncatula *pseudomolecules

Computational comparisons were made between the cowpea GSRs and available consensus ESTs (unigenes) from other legumes available at the Legume Information System (LIS) website [125]. Each legume EST was searched against the total cowpea GSRs dataset using tblastx with a cutoff value of 1e-8. We also compared the cowpea GSRs to a dataset of 16,954 unigenes with an average size of 709 nucleotides (7894 assemblies with of average size 859 nucleotides; 9060 singletons of average size 578 bp) derived from 42,000 ESTs. The ESTs were generated from two different libraries (a root library and a leaf/stem library) comprising material from four drought stressed and non-stressed cowpea cultivars (Dan Ila, a type II drought tolerant cultivar; Tvu11986, a type I drought tolerant cultivar; Tvu7778, a drought susceptible cultivar; and 12008D (Tvu9956), an advanced forage line with good feed quality and reported drought tolerance. The EST sequence data was kindly communicated to us by Drs. Sarah Hearne and Richard Bishop (IITA and ILRI, Nairobi, Kenya). A full description of the libraries and their generation is in preparation (Hearne S, personal communication). For this comparison both tblastx and blastn was used.

To locate cowpea GSRs on the *M. truncatula *chromosome-scale pseudomolecules [126] we employed tblastx with a threshold of 1e-5 and *Medicago truncatula *Gbrowse Mtr 1.0 pseudomolecule release.

### Identification of cowpea transcription factors and phylogenetic analysis of TF gene families

Homology searches (tblastn) of the cowpea GSRs were performed with the amino acid sequences of the DNA binding domains from each of the ten major ERF subgroups [see Additional file 5 and Additional file 6], representative WRKY domains from Groups IIa, IIb, IIc, IId, IIe and III and a N-terminal and C-terminal domain from Group I [see Additional file 7 and additional file 8], and the complete sequences of the *At CO*, *COL6 *and *COL9 *genes [see Additional file 9]. For each TF family, the GSRs recovered were assembled into contigs using a local web-based implementation of the Phrap program [36]. Each contig was then individually analyzed by blastx searches against the non-redundant protein database [120]. Sequences not containing the targeted TF domain under analysis were discarded. The minimum number of genes for each family was calculated based on the number of unique 5', 3', full-length and partial conserved domains present. Alignments of the predicted amino acid sequences of the conserved domains were carried out using ClustalW [127]-following removal of any intronic sequences. Phylogenetic trees were produced using the PHYLIP program using calculations based around the neighbor-joining method and are presented using *PhyloDraw *[128].

### Identification of simple sequence repeats (SSRs)

The presence of simple sequence repeats (SSRs) in each of the 263,425 cowpea GSRs was determined using the Tandem Repeats Finder program [129]. GSRs containing SSRs along with information on repeat size, composition and the primers for their amplification were parsed and loaded into relational tables for sorting, search, and joining [37].

## Authors' contributions

MPT conceived of the project and was responsible for directing all of the research activities. TWL participated in the database design, database system administration, and data analysis. XC performed the bioinformatics data analysis and web implementation. PJR, MTB, EC contributed to data analysis and phylogenetic analysis. FC performed the mapping of assemblies onto *M. truncatula *pseudomolecules. CDT assisted in data analysis can analysis of GSR mapping onto *M. truncatula *pseudomolecules. All authors have assisted in the writing of the manuscript and have read and approved the final submitted version of the manuscript.

## Supplementary Material

Additional file 1 Gene-enrichment calculation for methylation filtered versus unfiltered whole genome shotgun reads. Table showing the gene-enrichment calculation for DNA sequences taken from methylation filtered (MF) libraries prepared using GeneThresher^® ^technology and unfiltered (UF) libraries. Click here for file

Additional file 2 Distribution of read lengths in successful gene-space sequencing attempts. Table showing the distribution of different read length categories among all successful sequencing attempts of MF clones. Click here for file

Additional file 3 Distribution of cowpea GSR assemblies and singletons on the *M. truncatula *chromosome-scale pseudomolecules. Table shows the distribution of cowpea GSR assemblies and singletons that map by tblastx to the various *M. truncatula *chromosome-scale pseudomolecules. Click here for file

Additional file 4 Cowpea homologues of previously identified ERF genes in other plant species. Table showing the cowpea homologues of previously identified ERF genes that have been shown to regulate important agronomic traits in other plant species. Click here for file

Additional file 5 Amino acid sequences of conserved DNA binding domains used for the identification of cowpea ERFs. Table showing the amino acid sequences of conserved DNA binding domains used to identify cowpea ERFs in the GSR dataset using tbastn searches. Click here for file

Additional file 6 List of cowpea GSRs used in the determination of the phylogenetic relationships of predicted ERF genes in cowpea. Table listing the predicted cowpea ERF gene and the GSR identification number(s) for the sequence reads used in assembly of the binding domain used in the analysis. Click here for file

Additional file 7 Amino acid sequences of conserved DNA binding domains used for the identification of cowpea WRKY transcription factors. Table showing the amino acid sequences of conserved DNA binding domains used to identify cowpea WRKY transcription factors in the GSR dataset using tbastn searches. Click here for file

Additional file 8 List of cowpea GSR identification numbers and Genbank accession numbers of genes used in the determination of the phylogenetic relationships of predicted WRKY genes in cowpea. Table listing the predicted cowpea WRKY genes and the GSR identification number(s) for the sequence reads and Genbank accession numbers of genes used in assembly of the binding domain used in the analysis. Click here for file

Additional file 9 List of cowpea GSR identification numbers and Genbank accession numbers of genes used in the determination of the phylogenetic relationships of predicted *CONSTANS *and *CONSTANS*-like genes in cowpea. Table listing the predicted *CONSTANS *and *CONSTANS*-like genes in cowpea and the GSR identification number(s) for the sequence reads and Genbank accession numbers of genes used in assembly of the binding domain used in the analysis. Click here for file
