# Supplementary material for: Sequencing and analysis of the gene-rich space of cowpea
Source: BMC Genomics. 2008 Feb 27;9:103. doi: 10.1186/1471-2164-9-103 (PMC2279124; doi:10.1186/1471-2164-9-103)
Supplement: Additional file 1 — Gene-enrichment calculation for methylation filtered versus unfiltered whole genome shotgun reads. Table showing the gene-enrichment calculation for DNA sequences taken from methylation filtered (MF) libraries prepared using GeneThresher® technology and unfiltered (UF) libraries. [file 1471-2164-9-103-S1.doc]

**Additional file 1**

Gene-enrichment calculation for methylation filtered versus unfiltered whole genome shotgun reads.

Shown in the table below is the gene-enrichment calculation for DNA sequences taken from methylation filtered (MF) libraries prepared using GeneThresher® technology and unfiltered (UF) libraries.

MF library UF library

E-value # Hits to # Hits to Filter Power

# Seqs AT Ptns % Hits # Seqs AT Ptns % Hits

10e-5 766 316 41.25% 954 102 10.69% 3.9

10e-6 766 315 41.12% 954 102 10.69% 3.8

10e-7 766 302 39.43% 954 99 10.38% 3.8

10e-8 766 294 38.38% 954 96 10.06% 3.8

10e-9 766 283 36.95% 954 90 9.43% 3.9

10e-10 766 275 35.90% 954 86 9.01% 4.0

10e-11 766 266 34.73% 954 82 8.60% 4.0

10e-12 766 251 32.77% 954 76 7.97% 4.1

10e-13 766 242 31.59% 954 74 7.76% 4.1

10e-14 766 235 30.68% 954 69 7.23% 4.2

10e-15 766 227 29.63% 954 63 6.81% 4.3

10e-16 766 216 28.20% 954 62 6.50% 4.3

10e-17 766 206 26.89% 954 60 6.29% 4.3

10e-18 766 199 25.95% 954 59 6.12% 4.2

10e-19 766 193 25.20% 954 55 5.77% 4.4

10e-20 766 187 24.41% 954 54 5.66% 4.3

Median Value = 4.1
