# Supplementary material for: Sequencing and analysis of the gene-rich space of cowpea
Source: BMC Genomics. 2008 Feb 27;9:103. doi: 10.1186/1471-2164-9-103 (PMC2279124; doi:10.1186/1471-2164-9-103)
Supplement: Additional file 2 — Distribution of read lengths in successful gene-space sequencing attempts. Table showing the distribution of different read length categories among all successful sequencing attempts of MF clones. [file 1471-2164-9-103-S2.doc]

**Additional file 2**

Distribution of read lengths in successful gene-space sequencing attempts^a^.

Trimmed length (bp)

Low value High value Frequency

0 49 0

50 99 0

100 149 2073

150 199 2312

200 249 2757

250 299 3038

300 349 3679

350 399 4120

400 449 5303

450 499 7474

500 549 11566

550 599 23922

600 649 76961

650 699 97914

700 749 22135

750 799 170

800 849 1

850 899 0

Total 263425

^a^ Successful reads were greater than 100 bp in length and did not match DNA of extracellular (fungal, insect, bacterial or viral) and cytoplasmic (mitochondrion and chloroplast) origin when compared to available databases using blastx.
