# Supplementary material for: Sequencing and analysis of the gene-rich space of cowpea
Source: BMC Genomics. 2008 Feb 27;9:103. doi: 10.1186/1471-2164-9-103 (PMC2279124; doi:10.1186/1471-2164-9-103)
Supplement: Additional file 3 — Distribution of cowpea GSR assemblies and singletons on the M. truncatula chromosome-scale pseudomolecules. Table shows the distribution of cowpea GSR assemblies and singletons that map by tblastx to the various M. truncatula chromosome-scale pseudomolecules. [file 1471-2164-9-103-S3.doc]

**Additional file 3.**

Distribution of cowpea GSR assemblies and singletons on the *M. truncatula* chromosome-scale pseudomolecules.

Table shows the distribution of cowpea GSR assemblies and singletons that map by tblastx to the various *M. truncatula* chromosome-scale pseudomolecules.

| Pseudomolecule No. of Singletons No. of Assemblies |
| --- |
|  |
| chr1      2134       2815 chr4      2330       3211 chr5 2575 3718 chr2 2140 2907 chr3 3552 3624 chr6 832 865 chr7 2155 2973 chr8 2351 3110 chr0 844 852 |
|  |
| Total mapped 18913 24075 |
