# Supplementary material for: Sequencing and analysis of the gene-rich space of cowpea
Source: BMC Genomics. 2008 Feb 27;9:103. doi: 10.1186/1471-2164-9-103 (PMC2279124; doi:10.1186/1471-2164-9-103)
Supplement: Additional file 4 — Cowpea homologues of previously identified ERF genes in other plant species. Table showing the cowpea homologues of previously identified ERF genes that have been shown to regulate important agronomic traits in other plant species. [file 1471-2164-9-103-S4.doc]

**Additional file 4.**

Cowpea homologues of previously identified ERF genes in other plant species.

Group Gene Function Closest Homologue(s)

III *CBF1, DREB1A* freezing, drought, ERF104, ERF102,

and salt tolerance ERF110

III *TINY* growth regulation ERF4, ERF37

VII *CaPF1* freezing tolerance, ERF7, ERF9, ERF91

disease resistance

IX *ORCA3* jasmonic acid signaling, ERF85, ERF75

Secondary metabolism

IX *Pti4* disease resistance ERF3, ERF58
