# Supplementary material for: Sequencing and analysis of the gene-rich space of cowpea
Source: BMC Genomics. 2008 Feb 27;9:103. doi: 10.1186/1471-2164-9-103 (PMC2279124; doi:10.1186/1471-2164-9-103)
Supplement: Additional file 5 — Amino acid sequences of conserved DNA binding domains used for the identification of cowpea ERFs. Table showing the amino acid sequences of conserved DNA binding domains used to identify cowpea ERFs in the GSR dataset using tbastn searches. [file 1471-2164-9-103-S5.doc]

**Additional file 5.**

Amino acid sequences of conserved DNA binding domains used for the identification of cowpea ERFs.

Given are the amino acid sequences of conserved DNA binding domains used to identify cowpea ERFs in the GSR dataset using tbastn searches.

Group I

LYRGVRQRHWGKWVAEIRLPRNRTRLWLGTFDTAEEAALAYDKAAYKLRGDFARLNFP

Group II

RYKGIRMRKWGKWVAEIREPNKRSRIWLGSYKTAVAAARAYDTAVFYLRGPSARLNFP

Group III

IYRGVRQRNSGKWVSEVREPNKKTRIWLGTFQTAEMAARAHDVAALALRGRSACLNFA

Group IV

SFRGVRQRIWGKWVAEIREPNRGSRLWLGTFPTAQEAASAYDEAAKAMYGPLARLNFP

Group V

KFRGVRQRHWGSWVAEIRHPLLKRRIWLGTFETAEEAARAYDEAAVLMSGRNAKTNFP

Group VI

KFRGVRQRPWGKWAAEIRDPSRRVRVWLGTFDTAEEAAIVYDNAAIQLRGPNAELNFP

Group VII

VYRGIRKRPWGKWAAEIRDPRKGVRVWLGTFNTAEEAAMAYDVAAKQIRGDKAKLNFP

Group VIII

RFLGVRRRPWGRYAAEIRDPTTKERHWLGTFDTAEEAALAYDRAARSMRGTRARTNFV

Group IX

HYRGVRQRPWGKFAAEIRDPAKNGARVWLGTFETAEDAALAYDRAAFRMRGSRALLNFP

Group X

KYRGVRQRPWGKWAAEIRDPHKATRVWLGTFETAEAAARAYDAAALRFRGSKAKLNFP

Group VI-L

KPVGVRQRKWGKWAAEIRHPITKVRTWLGTYETLEQAADAYATKKLAFDALAAATSAA

Group XB-L

KHKGVRKKPSGKWAAEIWDPSLKVRRWLGTFPTAEMAAKAYNDAAAEFVGRRSARRGT
