# Supplementary material for: Sequencing and analysis of the gene-rich space of cowpea
Source: BMC Genomics. 2008 Feb 27;9:103. doi: 10.1186/1471-2164-9-103 (PMC2279124; doi:10.1186/1471-2164-9-103)
Supplement: Additional file 6 — List of cowpea GSRs used in the determination of the phylogenetic relationships of predicted ERF genes in cowpea. Table listing the predicted cowpea ERF gene and the GSR identification number(s) for the sequence reads used in assembly of the binding domain used in the analysis. [file 1471-2164-9-103-S6.doc]

**Additional file 6.**

List of cowpea GSRs used in the determination of the phylogenetic relationships of predicted ERF genes in cowpea.

Each putative ERF gene identified from the cowpea gene-space sequence is listed using the abbreviation Vu followed by the gene family member designation. Below each gene name is listed the GSR identification number(s) for the sequence reads used in assembly of the binding domain used in the analysis.

VuERF1.

962_34_14519358_5489_45019_030.ab1

VuERF2.

962_288_14618067_5489_46973_070.ab1

VuERF3.

962_78_14535692_5489_45331_093.ab1

VuERF4.

962_280_14614779_16654_46918_070.ab1

VuERF5.

962_335_14636632_16654_47374_043.ab1

VuERF6.

962_405_14672311_5489_48288_092.ab1

VuERF7.

962_280_14614731_5489_46914_072.ab1

VuERF8.

962_255_14604027_16654_46741_078.ab1

VuERF9.

962_64_14530533_5489_45250_083.ab1

VuERF10.

962_257_14604949_5489_46751_023.ab1
962_139_14559432_5489_45852_075.ab1

VuERF11.

962_144_14561284_5489_45895_095.ab1
962_150_14563714_16654_45951_010.ab1

VuERF12.

962_292_14619668_5489_47011_057.ab1
962_408_14673680_5489_48247_001.ab1

VuERF13.

962_326_14633067_16654_47284_080.ab1
962_419_14677853_5489_48336_085.ab1

VuERF14.

962_138_14559130_16654_45848_008.ab1
962_32_14518716_5489_44933_055.ab1

VuERF15.

962_116_14550810_16654_45697_068.ab1
962_130_14556122_5489_45781_006.ab1

VuERF16.

962_416_14676423_16654_48306_032.ab1
962_104_14545718_5489_45591_060.ab1

VuERF17.

962_205_14585096_5489_46409_001.ab1
962_62_14529629_5489_45194_025.ab1

VuERF18.

962_423_14679142_16654_48363_064.ab1
962_423_14679142_5489_48359_064.ab1

VuERF19.

962_21_14514370_5489_44926_046.ab1
962_177_14574053_5489_46111_095.ab1

VuERF20.

962_134_14557634_16654_45817_072.ab1
962_322_14631673_16654_47260_073.ab1

VuERF21.

962_372_14651203_16654_48213_040.ab1
962_96_14542780_16654_45535_021.ab1

VuERF22.

962_239_14597844_5489_46531_031.ab1
962_250_14602264_16654_47993_039.ab1

VuERF23.

962_247_14600947_5489_46667_046.ab1
962_348_14642075_16654_47507_004.ab1

VuERF24.

962_7_14508832_16654_44903_045.ab1
962_260_14606252_16654_48145_049.ab1

VuERF25.

962_151_14564154_5489_45955_040.ab1
962_287_14617415_16654_46992_060.ab1

VuERF26.

962_376_14652767_16654_47678_056.ab1
962_49_14524791_5489_45082_052.ab1

VuERF27.

962_143_14561070_5489_48036_088.ab1
962_236_14596838_5489_48202_026.ab1

VuERF28.

962_231_14594886_5489_46548_092.ab1
962_116_14550643_16654_45696_074.ab1

VuERF29.

962_50_14525158_16654_45149_084.ab1
962_315_14628724_5489_47199_081.ab1

VuERF30.

962_333_14635930_16654_47356_008.ab1
962_416_14676457_5489_48304_079.ab1

VuERF31.

962_378_14653666_5489_47696_002.ab1
962_68_14531927_16654_45230_090.ab1

VuERF32.

962_101_14544757_5489_45556_051.ab1
962_309_14626296_16654_47153_069.ab1

VuERF33.

962_275_14612770_5489_46875_010.ab1
962_157_14566403_5489_46165_010.ab1

VuERF34.

962_257_14605028_5489_46754_051.ab1
962_159_14567379_5489_45980_066.ab1

VuERF35.

962_180_14575336_16654_46130_041.ab1
962_282_14615554_5489_46931_006.ab1

VuERF36.

962_252_14602872_5489_46711_077.ab1
962_283_14615930_16654_46941_070.ab1

VuERF37.

962_171_14571904_5489_46050_039.ab1
962_171_14571904_16654_46054_039.ab1

VuERF38.

962_174_14573104_5489_46075_037.ab1
962_130_14556218_5489_45781_002.ab1

VuERF39.

962_296_14621219_16654_47047_010.ab1
962_294_14620385_5489_47028_043.ab1

VuERF40.

962_281_14615052_16654_46925_025.ab1
962_308_14625785_16654_47148_043.ab1

VuERF41.

962_43_14522192_16654_45001_047.ab1
962_307_14625294_5489_47133_096.ab1

VuERF42.

962_319_14629902_5489_47226_096.ab1
962_394_14659673_5489_47968_047.ab1

VuERF43.

962_405_14672451_5489_48288_070.ab1
962_366_14648839_16654_47641_092.ab1

VuERF44.

962_64_14530375_5489_45250_026.ab1
962_75_14534732_5489_45298_085.ab1

VuERF45.

962_307_14625325_5489_47134_029.ab1
962_405_14672465_5489_48227_037.ab1

VuERF46.

962_142_14560758_5489_45878_086.ab1
962_278_14613825_16654_46904_013.ab1

VuERF47.

962_245_14600218_16654_46648_012.ab1
962_181_14575634_5489_46150_078.ab1
962_27_14516809_5489_45045_007.ab1

VuERF48.

962_244_14599920_5489_46639_073.ab1
962_139_14559365_5489_45855_095.ab1
962_70_14532831_5489_45366_052.ab1

VuERF49.

962_419_14677688_5489_48335_011.ab1
962_274_14612576_5489_46867_001.ab1
962_53_14526052_16654_45119_029.ab1

VuERF50.

962_221_14591126_16654_46463_024.ab1
962_221_14590940_16654_46463_063.ab1
962_230_14594611_5489_46539_038.ab1

VuERF51.

962_101_14544597_16654_45567_089.ab1
962_204_14584689_16654_46407_003.ab1
962_70_14532812_16654_45371_085.ab1

VuERF52.

962_298_14621945_5489_47062_043.ab1
962_220_14590630_5489_48091_060.ab1
962_178_14574655_16654_46121_086.ab1

VuERF53.

962_343_14640236_5489_47458_049.ab1
962_362_14647222_5489_47553_062.ab1
962_32_14518577_5489_47954_077.ab1

VuERF54.

962_178_14574721_5489_46119_067.ab1
962_210_14586842_16654_47985_074.ab1
962_104_14545889_16654_45650_067.ab1

VuERF55.

962_108_14547681_16654_45621_037.ab1
962_27_14516794_16654_44920_040.ab1
962_80_14536530_16654_47978_074.ab1

VuERF56.

962_48_14524375_16654_45084_020.ab1
962_311_14627047_5489_47165_088.ab1
962_21_14514609_16654_45774_035.ab1

VuERF57.

962_379_14653812_16654_47704_027.ab1
962_378_14653630_5489_47696_052.ab1
962_235_14596467_5489_46578_074.ab1

VuERF58.

962_294_14620348_16654_47031_093.ab1
962_222_14591658_16654_46471_034.ab1
962_332_14635445_16654_47351_093.ab1

VuERF59.

962_58_14528265_5489_45170_033.ab1
962_286_14616842_16654_46966_080.ab1
962_122_14553081_16654_45720_035.ab1

VuERF60.

962_297_14621646_16654_47055_088.ab1
962_116_14550743_5489_45692_086.ab1
962_198_14582162_16654_46308_078.ab1

VuERF61.

962_185_14577157_16654_46221_029.ab1
962_29_14517471_16654_45056_060.ab1
962_178_14574497_5489_46117_043.ab1
962_128_14555247_5489_47927_058.ab1

VuERF62.

962_351_14642953_16654_47541_079.ab1
962_275_14612802_16654_46877_040.ab1
962_250_14602087_5489_47977_096.ab1
962_137_14558692_16654_45842_091.ab1

VuERF63.

962_105_14546262_5489_45589_020.ab1
962_104_14545903_5489_45590_020.ab1
962_353_14643870_5489_47598_090.ab1
962_376_14652916_5489_47675_081.ab1

VuERF64.

962_96_14542704_5489_45519_007.ab1
962_202_14583898_16654_48078_004.ab1
962_202_14583615_16654_48077_032.ab1
962_156_14566139_5489_48038_004.ab1

VuERF65.

962_211_14587093_5489_48024_031.ab1
962_147_14562408_16654_45923_079.ab1
962_406_14672900_16654_48238_051.ab1
962_94_14541932_5489_45491_089.ab1

VuERF66.
962_271_14611217_16654_48181_041.ab1
962_164_14568938_5489_46184_080.ab1
962_355_14644673_5489_47651_039.ab1
962_345_14640814_5489_47489_058.ab1
962_159_14567111_5489_45978_060.ab1

VuERF67

962_384_14655733_16654_47764_027.ab1

VuERF68

962_146_14562187_5489_45914_042.ab1

VuERF69

962_120_14552169_5489_45700_041.ab1

VuERF70

962_385_14656371_16654_47740_066.ab1

VuERF71

962_243_14599447_16654_46634_094.ab1

VuERF72

962_309_14626296_5489_47149_069.ab1

VuERF73

962_153_14565057_16654_46229_001.ab1

VuERF74

962_97_14542976_5489_45525_045.ab1

VuERF75

962_359_14646085_16654_47310_029.ab1

VuERF76

962_375_14652283_5489_47666_044.ab1

VuERF77

962_261_14606312_5489_46775_013.ab1

VuERF78

962_137_14558724_5489_45838_025.ab1

VuERF79

962_211_14587362_5489_48022_036.ab1

VuERF80

962_104_14545941_5489_45648_081.ab1

VuERF81

962_379_14653867_16654_47705_042.ab1

VuERF82

962_214_14588435_16654_48074_008.ab1

VuERF83

962_201_14583501_16654_46380_053.ab1

VuERF84

962_259_14605690_16654_46763_008.ab1

VuERF85

962_182_14575957_5489_46159_031.ab1

VuERF86

962_286_14617274_5489_46964_050.ab1

VuERF87

962_320_14630593_16654_47246_067.ab1

VuERF88

962_374_14651834_5489_48013_078.ab1

VuERF89

962_418_14677525_5489_48326_017.ab1

VuERF90

962_217_14589512_16654_48123_009.ab1

VuERF91

962_34_14519482_5489_44934_040.ab1

VuERF92

962_421_14678543_5489_48352_056.ab1

VuERF93

962_161_14568108_5489_45993_017.ab1

VuERF94

962_69_14532328_16654_45275_073.ab1

VuERF95

962_405_14672386_5489_48225_008.ab1

VuERF96

962_286_14616923_5489_46963_012.ab1

VuERF97

962_126_14554375_5489_45750_030.ab1

VuERF98

962_284_14616385_5489_46949_067.ab1

VuERF99

962_350_14642830_16654_47534_054.ab1

VuERF100

962_376_14652654_5489_47677_092.ab1

VuERF101

962_177_14574165_5489_46109_057.ab1

VuERF102

962_103_14545256_5489_45574_045.ab1

VuERF103

962_293_14620209_16654_47026_003.ab1

VuERF104

962_419_14677569_16654_48338_015.ab1

VuERF105

962_103_14545447_16654_45583_022.ab1

VuERF106

962_231_14595050_16654_46550_068.ab1

VuERF107

962_397_14661005_5489_47855_087.ab1

VuERF108

962_45_14522979_5489_45007_014.ab1

VuERF109

962_306_14624980_5489_47131_093.ab1

VuERF110

962_92_14541079_5489_45470_028.ab1

VuERF111

962_404_14671944_16654_48278_075.ab1
