# Supplementary material for: Sequencing and analysis of the gene-rich space of cowpea
Source: BMC Genomics. 2008 Feb 27;9:103. doi: 10.1186/1471-2164-9-103 (PMC2279124; doi:10.1186/1471-2164-9-103)
Supplement: Additional file 7 — Amino acid sequences of conserved DNA binding domains used for the identification of cowpea WRKY transcription factors. Table showing the amino acid sequences of conserved DNA binding domains used to identify cowpea WRKY transcription factors in the GSR dataset using tbastn searches. [file 1471-2164-9-103-S7.doc]

**Additional file 7.**

Amino acid sequences of conserved DNA binding domains used for the identification of cowpea WRKY transcription factors.

Given below are the amino acid sequences of conserved DNA binding domains used to identify cowpea WRKY transcription factors in the GSR dataset using tbastn searches.

I N-terminal

DGYNWRKYGQKLVKGNEFVRSYYRCTHPNCKAKKQLERSAGGQVVDTVYFGEHDH

I C-terminal

DGYRWRKYGQKSVKGSPYPRSYYRCSSPGCPVKKHVERSSHDTKLLITTYEGKHDH

IIa

DGYQWRKYGQKVTRDNPSPRAYFKCACAPSCSVKKKVQRSVEDQSVLVATYEGEHNH

IIb

DGCQWRKYGQKMAKGNPCPRAYYRCTMATGCPVRKQVQRCAEDRSILITTYEGNHNH

IIc

DDGYRWRKYGQKVVKNTQHPRSYYRCTQDKCRVKKRVERLADDPRMVITTYEGRHLH

IId

DEFSWRKYGQKPIKGSPHPRGYYKCSSVRGCPARKHVERALDDAMMLIVTYEGDHNH

IIe

DVWAWRKYGQKPIKGSPYPRGYYRCSTSKGCLARKQVERNRSDPKMFIVTYTAEHNH

III

DDGFSWRKYGQKDILGAKFPRGYYRCTYRKSQGCEATKQVQRSDENQMLLEISYRGIHSC

WIZZ

KDGYQWRKYGQKVTRDNPSPRAYFRCSFAPGCPVKKKVQRSIEDQSVVVATYEGEHNH

AtWRKY1 N-terminal

DGYNWRKYGQKQVKGSENPRSYYKCTFPNCPTKKKVERNLDGHITEIVYKGNHNH

AtWRKY1 C-terminal

DGYRWRKYGQKVAKGNPNPRSYYKCTFTGCPVRKHVERASHDLRAVITTYEGKHNH

AtWRKY2 N-terminal

DGYNWRKYGQKQVKGSENPRSYYKCTFPNCPTKKKVERSLDGQITEIVYKGNHNH

AtWRKY2 C-terminal

DGYRWRKYGQKVVKGNPNPRGYYKCTSPGCPVRKHVERASQDIRSVITTYEGKHNH

AtWRKY3

DEYSWRKYGQKPIKGSPYPRGYYKCSSVRGCPARKHVERAMDDPAMLIVTYEGEHRH

SUSIBA2 N-terminal

DGYNWRKYGQKHVKGSENPRSYYKCTHPNCEVKKLLERAVDGLITEVVYKGRHNH

SUSIBA2 C-terminal

DGYRWRKYGQKVVKGNPNPRSYYKCTSTGCPVRKHVERASHDPKSVITTYEGKHNH

AtWRKY4 N-terminal

DGYNWRKYGQKQVKGSEYPRSYYKCTHPNCPVKKKVERSHEGHITEIIYKGAHNH

AtWRKY4 C-terminal

DGYRWRKYGQKVVKGNPNPRSYYKCTSAGCNVRKHVERASHDLKSVITTYEGKHNH

ACRE126

DGCQWRKYGQKISRGNPCPRSYYRCSVAPLCPVRKQVQRCVEDMSVLITTYEGTHNH
