# Supplementary material for: Sequencing and analysis of the gene-rich space of cowpea
Source: BMC Genomics. 2008 Feb 27;9:103. doi: 10.1186/1471-2164-9-103 (PMC2279124; doi:10.1186/1471-2164-9-103)
Supplement: Additional file 8 — List of cowpea GSR identification numbers and Genbank accession numbers of genes used in the determination of the phylogenetic relationships of predicted WRKY genes in cowpea. Table listing the predicted cowpea WRKY genes and the GSR identification number(s) for the sequence reads and Genbank accession numbers of genes used in assembly of the binding domain used in the analysis. [file 1471-2164-9-103-S8.doc]

**Additional file 8.**

List of cowpea GSR identification numbers and Genbank accession numbers of genes used in the determination of the phylogenetic relationships of predicted WRKY genes in cowpea.

Each putative WRKY gene identified from the cowpea gene-space sequence is listed using the abbreviation Vu followed by the gene family member designation. Below each gene name is listed the GSR identification number(s) for the sequence reads used in assembly of the binding domain used in the analysis. Arabidopsis genes used in the comparison are designated by At followed by the gene member number and Genbank accession number.

VuWRKY1.

962_52_14525990_5489_45109_050.ab1

VuWRKY2.

962_158_14566770_5489_46017_042.ab1

VuWRKY3.

962_341_14639150_16654_47468_030.ab1

VuWRKY4.

962_269_14610296_5489_46844_015.ab1

VuWRKY5.

962_168_14570983_5489_46037_092.ab1
962_394_14659806_16654_47969_090.ab1

VuWRKY6.

962_234_14596229_5489_46606_083.ab1
962_126_14554474_16654_45751_042.ab1

VuWRKY7.

962_54_14526756_5489_45093_049.ab1
962_54_14526756_16654_45101_049.ab1

VuWRKY8.

962_76_14534961_16654_45340_043.ab1
962_167_14570084_16654_46030_063.ab1

VuWRKY9.

962_146_14562023_5489_45936_064.ab1
962_387_14657058_16654_47769_044.ab1

VuWRKY10.

962_218_14589988_16654_48150_087.ab1
962_208_14586209_16654_46423_035.ab1

VuWRKY11.

962_178_14574565_5489_46119_025.ab1
962_251_14602802_16654_46701_066.ab1

VuWRKY12.

962_64_14530234_5489_45249_048.ab1
962_314_14628263_5489_47190_052.ab1

VuWRKY13.

962_95_14542357_5489_45518_055.ab1
962_305_14624826_16654_47122_036.ab1

VuWRKY14.

962_379_14653834_5489_47698_010.ab1
962_349_14642399_5489_47523_056.ab1

VuWRKY15.

962_201_14583533_5489_48053_083.ab1
962_144_14561514_16654_45899_038.ab1

VuWRKY16.

962_369_14650110_5489_47588_086.ab1
962_342_14639759_5489_47479_054.ab1

VuWRKY17.

962_232_14595418_16654_46558_004.ab1
962_242_14599284_5489_46621_019.ab1

VuWRKY18.

962_186_14577768_16654_46258_067.ab1
962_330_14634696_16654_47328_075.ab1

VuWRKY19.

962_178_14574647_16654_46121_054.ab1
962_188_14578344_16654_46274_075.ab1

VuWRKY20.

962_321_14631484_5489_47247_081.ab1
962_98_14543510_5489_45529_056.ab1

VuWRKY21.

962_302_14623613_16654_47097_085.ab1
962_388_14657532_16654_47777_023.ab1

VuWRKY22.

962_145_14561898_5489_45903_038.ab1
962_100_14544335_16654_45564_086.ab1

VuWRKY23.

962_299_14622467_5489_47070_006.ab1
962_385_14656174_16654_47739_058.ab1

VuWRKY24.

962_48_14524101_5489_45076_095.ab1
962_43_14522444_16654_45001_085.ab1

VuWRKY26.

962_55_14526972_5489_45145_055.ab1
962_203_14584151_5489_48056_058.ab1

VuWRKY27.

962_404_14671990_16654_48278_058.ab1
962_309_14626120_5489_47151_045.ab1
962_427_14680640_5489_48391_015.ab1

VuWRKY28.

962_53_14526280_16654_45121_069.ab1
962_45_14523230_16654_45009_052.ab1
962_276_14613056_5489_46883_013.ab1

VuWRKY29.

962_121_14552715_16654_45714_004.ab1
962_322_14631628_5489_47255_091.ab1
962_400_14670551_16654_48191_054.ab1

VuWRKY30.

962_140_14559950_16654_45864_022.ab1
962_52_14525963_5489_45108_034.ab1
962_89_14540065_5489_47879_005.ab1

VuWRKY31.

962_422_14678933_16654_48343_087.ab1
962_422_14678933_5489_48344_087.ab1
962_266_14608261_16654_46812_029.ab1

VuWRKY32.

962_274_14612457_5489_46866_005.ab1
962_69_14532469_16654_45276_051.ab1
962_75_14534695_5489_45297_022.ab1

VuWRKY33.

962_277_14613595_5489_46892_040.ab1
962_295_14620959_16654_47040_020.ab1
962_294_14620568_5489_47027_003.ab1
962_295_14620959_5489_47036_020.ab1

VuWRKY34.

962_222_14591398_5489_46465_060.ab1
962_100_14544259_5489_45550_072.ab1
962_259_14605584_5489_46761_077.ab1
962_50_14525112_5489_45139_005.ab1

VuWRKY35.

962_335_14636796_16654_47372_019.ab1
962_137_14558583_16654_45843_032.ab1
962_18_14513271_5489_44931_056.ab1
962_405_14672228_16654_48230_063.ab1

VuWRKY36.

962_184_14576721_16654_46212_015.ab1
962_168_14571058_5489_46036_008.ab1
962_122_14553041_16654_45720_069.ab1
962_184_14576721_5489_46208_015.ab1
962_328_14634147_5489_47434_068.ab1
962_376_14652874_5489_47677_002.ab1

VuWRKY37.

962_133_14557226_5489_45803_072.ab1
962_160_14567389_5489_45986_031.ab1
962_427_14680775_16654_48398_060.ab1
962_427_14680871_16654_48398_056.ab1
962_124_14553900_16654_45735_049.ab1
962_265_14607859_5489_46818_046.ab1
962_160_14567389_16654_45990_031.ab1

VuWRKY38.

962_187_14578066_5489_46264_008.ab1
962_327_14633526_16654_47296_094.ab1
962_117_14551235_16654_47909_034.ab1
962_247_14601198_5489_46666_084.ab1
962_167_14570171_5489_46027_012.ab1
962_151_14564126_16654_45957_024.ab1
962_327_14633526_5489_47292_094.ab1
962_52_14525863_5489_45108_022.ab1

VuWRKY39

962_216_14589089_16654_48065_043.ab1

VuWRKY41

962_231_14595098_16654_46550_066.ab1

VuWRKY42

962_282_14615424_5489_46931_075.ab1

VuWRKY43

962_134_14557736_5489_45811_001.ab1

VuWRKY44

962_307_14625606_5489_47135_084.ab1

VuWRKY45

962_228_14593791_5489_46514_024.ab1

VuWRKY46

962_177_14574154_16654_46112_010.ab1

VuWRKY47

962_187_14578193_16654_48154_033.ab1

VuWRKY48

962_116_14550593_5489_45692_075.ab1

VuWRKY49

962_157_14566278_5489_46166_096.ab1

VuWRKY50

962_142_14560803_16654_45884_068.ab1

VuWRKY51

962_48_14524437_16654_45084_081.ab1

VuWRKY52

962_280_14614750_16654_46919_056.ab1

VuWRKY53

962_343_14640027_5489_47457_074.ab1

VuWRKY54

962_28_14517171_5489_45046_008.ab1

VuWRKY55

962_309_14626120_16654_47155_045.ab1

VuWRKY56

962_314_14628263_16654_47194_052.ab1

VuWRKY57

962_45_14523264_16654_45009_001.ab1

VuWRKY58

962_170_14571507_16654_45312_074.ab1

VuWRKY59

962_380_14654217_16654_47711_009.ab1

VuWRKY60

962_308_14625810_16654_47145_042.ab1

VuWRKY61

962_315_14628479_5489_47200_060.ab1

VuWRKY62

962_424_14679521_16654_48372_047.ab1

VuWRKY63

962_167_14570183_5489_46027_060.ab1

VuWRKY64

962_234_14596099_5489_46604_040.ab1

VuWRKY65

962_125_14554157_5489_45742_023.ab1

VuWRKY66

962_247_14601138_5489_46666_038.ab1

VuWRKY67

962_111_14548919_16654_45645_082.ab1

VuWRKY68

962_350_14642932_5489_47530_081.ab1

VuWRKY69

962_266_14608433_16654_46814_037.ab1

VuWRKY70

962_65_14530672_16654_45237_077.ab1

VuWRKY71

962_187_14578119_16654_46269_022.ab1

VuWRKY72

962_117_14550883_5489_47903_080.ab1

VuWRKY73

962_281_14615120_16654_46927_007.ab1

VuWRKY74

962_328_14634147_16654_47438_068.ab1

VuWRKY75

962_255_14604069_16654_46741_059.ab1

VuWRKY76

962_406_14672920_5489_48232_033.ab1

VuWRKY77

962_261_14606582_5489_46777_020.ab1

VuWRKY78

962_200_14583013_16654_48035_025.ab1

VuWRKY79

962_314_14628055_5489_47192_094.ab1

VuWRKY80

962_29_14517579_16654_45058_008.ab1

VuWRKY81

962_138_14559191_16654_45849_054.ab1

AtWRKY1

At2g04880

AtWRKY2

At5g56270

AtWRKY6

At4g26640

AtWRKY7

At4g24240

AtWRKY8

At5g46350

AtWRKY11

At4g31550

AtWRKY13

At4g39410

AtWRKY14

At1g30650

AtWRKY18

At4g31795

AtWRKY19

At4g12020

AtWRKY20

At4g26640

AtWRKY21

At2g30590

AtWRKY22

At4g01250

AtWRKY24

At4g30935

AtWRKY27

At5g52830

AtWRKY28

At4g18170

AtWRKY30

At5g24110

AtWRKY31

At4g01250

AtWRKY33

At2g38470

AtWRKY39

At3g04670

AtWRKY40

At1g80840

AtWRKY43

At2g46130

AtWRKY44

At2g37260

AtWRKY48

At5g49520

AtWRKY50

At5g26170

AtWRKY52

At5g45270

AtWRKY60

At2g25000

AtWRKY69

At3g58710

AtWRKY70

At3g56400

AtWRKY71

At1g29860

**Supplemental Table S9.** Supplemental Table S9**.** List of cowpea GSRs and Genbank accession numbers of genes used for determination of phylogenetic relationships of *CONSTANS* and *CONSTANS*-like genes in cowpea.

Each putative *CONSTANS* and *CONSTANS*-like gene identified from the cowpea gene-space sequence is listed using the abbreviation Vu followed by the gene family member designation. Below the gene name is the identification numbers of the GSRs that were used to form the domain used in the analysis. Arabidopsis genes used in the comparison are designated by the prefix At and are followed by the Genbank accession number of that gene; *M. truncatula* genes are designated by the prefix Mt, *Pisum sativum* genes by the prefix Ps, barley by the prefix Hv, and rice genes by the prefix Hd.

VuCOL1

962_217_14589687_16654_48128_020.ab1
962_274_14612445_16654_46872_055.ab1
962_187_14578210_5489_46264_002.ab1

VuCOL2

962_257_14604732_5489_46754_031.ab1

VuCOL3

962_295_14620893_5489_47038_055.ab1
962_259_14605658_16654_46763_074.ab1
962_277_14613581_5489_46890_087.ab1
962_78_14535742_16654_45343_092.ab1
962_295_14620893_16654_47042_055.ab1
962_398_14661475_5489_47863_036.ab1
962_128_14555100_5489_47926_063.ab1

VuCOL4

962_31_14519670_5489_44932_063.ab1
962_324_14632525_16654_47268_021.ab1
962_61_14529096_5489_45200_015.ab1
962_61_14529096_16654_45210_015.ab1
962_324_14632525_5489_47264_021.ab1
962_181_14575850_5489_46148_068.ab1
962_53_14526046_5489_45113_096.ab1

VuCOL5

962_145_14561863_5489_45904_088.ab1
962_238_14597534_16654_46574_028.ab1

VuCOL6

962_46_14523547_16654_45070_072.ab1
962_424_14679860_5489_48367_049.ab1

VuCOL7

962_43_14522355_5489_44994_008.ab1
962_160_14567611_5489_45988_040.ab1

VuCOL8

962_253_14603257_16654_46724_077.ab1
962_93_14541404_16654_45485_095.ab1

VuCOL9

962_392_14658892_5489_47813_095.ab1
962_151_14564255_16654_45960_052.ab1
962_258_14605144_16654_46709_047.ab1

VuCOL10

962_257_14605080_5489_46754_065.ab1
962_360_14646514_5489_47451_012.ab1

VuCOL11

962_193_14580323_5489_46313_010.ab1

VuCOL12

962_96_14542675_5489_45520_074.ab1

VuCOL13

962_272_14611827_16654_46859_066.ab1
962_320_14630321_5489_47240_045.ab1

VuCOL14

962_55_14526784_16654_45157_079.ab1

VuCOL15

962_123_14553325_5489_45724_057.ab1

VuCOL16

962_2_14465892_16654_44877_001.ab1

VuCOL17

962_46_14523524_5489_45059_087.ab1

VuCOL18

962_163_14568579_5489_46004_080.ab1

VuCOL19

962_340_14638719_16654_47431_032.ab1

VuCOL20

962_200_14582916_16654_46371_029.ab1

VuCOL21

962_14_14511850_16654_44910_087.ab1
962_256_14604510_16654_46747_090.ab1
962_324_14632523_5489_47264_006.ab1
962_278_14613889_16654_46904_075.ab1

VuCOL22

962_153_14564733_5489_46225_063.ab1

VuCOL23

962_332_14635549_5489_47345_023.ab1

MtCOL1

AC146745_15.2

MtCOL2

CR954188_1.1

MtCOL3

CR962137_2.1

MtCOL4

AC127169_10.1

PsCOLa

AY830921

PsCOLb

AY830922

Hd1

BAB17628

Hd3a

BAB61030

Hd6

ABB17669

HvCOL1

AAM74063

HvCOL2

AAM74065

HvCOL3

AAM74068

HvCOL4

AAM74070

HvCOL6

AAL99267

CONSTANS

AT5G15840

AtCOL1

AT5G15850

AtCOL2

AT3G02380

AtCOL3

AT2G24790

AtCOL4

AT5G24930

AtCOL5

AT5G5660

AtCOL6

AT1G25440

AtCOL7

AT1G73870

AtCOL8

AT1G49130

AtCOL9

AT3G07650

AtCOL10

AT5G48250

AtCOL11

AT4G15250

AtCOL12

AT3G21880

AtCOL13

AT2G47890

AtCOL14

AT2G33500

AtCOL15

AT1G28050

AtCOL16

AT1G68520
