# Supplementary material for: Sequencing and analysis of the gene-rich space of cowpea
Source: BMC Genomics. 2008 Feb 27;9:103. doi: 10.1186/1471-2164-9-103 (PMC2279124; doi:10.1186/1471-2164-9-103)
Supplement: Additional file 9 — List of cowpea GSR identification numbers and Genbank accession numbers of genes used in the determination of the phylogenetic relationships of predicted CONSTANS and CONSTANS-like genes in cowpea. Table listing the predicted CONSTANS and CONSTANS-like genes in cowpea and the GSR identification number(s) for the sequence reads and Genbank accession numbers of genes used in assembly of the binding domain used in the analysis. [file 1471-2164-9-103-S9.doc]

**Additional file 9.**

List of cowpea GSR identification numbers and Genbank accession numbers of genes used in the determination of the phylogenetic relationships of predicted *CONSTANS* and *CONSTANS*-like genes in cowpea.

Each putative *CONSTANS* and *CONSTANS*-like gene identified from the cowpea gene-space sequence is listed using the abbreviation Vu followed by the gene family member designation. Below each gene name is listed the GSR identification number(s) for the sequence reads used in assembly of the binding domain used in the analysis. Arabidopsis genes used in the comparison are designated by the prefix At and are followed by the Genbank accession number of that gene; *M. truncatula* genes are designated by the prefix Mt, *Pisum sativum* genes by the prefix Ps, barley by the prefix Hv, and rice genes by the prefix Hd.

VuCOL1

962_217_14589687_16654_48128_020.ab1
962_274_14612445_16654_46872_055.ab1
962_187_14578210_5489_46264_002.ab1

VuCOL2

962_257_14604732_5489_46754_031.ab1

VuCOL3

962_295_14620893_5489_47038_055.ab1
962_259_14605658_16654_46763_074.ab1
962_277_14613581_5489_46890_087.ab1
962_78_14535742_16654_45343_092.ab1
962_295_14620893_16654_47042_055.ab1
962_398_14661475_5489_47863_036.ab1
962_128_14555100_5489_47926_063.ab1

VuCOL4

962_31_14519670_5489_44932_063.ab1
962_324_14632525_16654_47268_021.ab1
962_61_14529096_5489_45200_015.ab1
962_61_14529096_16654_45210_015.ab1
962_324_14632525_5489_47264_021.ab1
962_181_14575850_5489_46148_068.ab1
962_53_14526046_5489_45113_096.ab1

VuCOL5

962_145_14561863_5489_45904_088.ab1
962_238_14597534_16654_46574_028.ab1

VuCOL6

962_46_14523547_16654_45070_072.ab1
962_424_14679860_5489_48367_049.ab1

VuCOL7

962_43_14522355_5489_44994_008.ab1
962_160_14567611_5489_45988_040.ab1

VuCOL8

962_253_14603257_16654_46724_077.ab1
962_93_14541404_16654_45485_095.ab1

VuCOL9

962_392_14658892_5489_47813_095.ab1
962_151_14564255_16654_45960_052.ab1
962_258_14605144_16654_46709_047.ab1

VuCOL10

962_257_14605080_5489_46754_065.ab1
962_360_14646514_5489_47451_012.ab1

VuCOL11

962_193_14580323_5489_46313_010.ab1

VuCOL12

962_96_14542675_5489_45520_074.ab1

VuCOL13

962_272_14611827_16654_46859_066.ab1
962_320_14630321_5489_47240_045.ab1

VuCOL14

962_55_14526784_16654_45157_079.ab1

VuCOL15

962_123_14553325_5489_45724_057.ab1

VuCOL16

962_2_14465892_16654_44877_001.ab1

VuCOL17

962_46_14523524_5489_45059_087.ab1

VuCOL18

962_163_14568579_5489_46004_080.ab1

VuCOL19

962_340_14638719_16654_47431_032.ab1

VuCOL20

962_200_14582916_16654_46371_029.ab1

VuCOL21

962_14_14511850_16654_44910_087.ab1
962_256_14604510_16654_46747_090.ab1
962_324_14632523_5489_47264_006.ab1
962_278_14613889_16654_46904_075.ab1

VuCOL22

962_153_14564733_5489_46225_063.ab1

VuCOL23

962_332_14635549_5489_47345_023.ab1

MtCOL1

AC146745_15.2

MtCOL2

CR954188_1.1

MtCOL3

CR962137_2.1

MtCOL4

AC127169_10.1

PsCOLa

AY830921

PsCOLb

AY830922

Hd1

BAB17628

Hd3a

BAB61030

Hd6

ABB17669

HvCOL1

AAM74063

HvCOL2

AAM74065

HvCOL3

AAM74068

HvCOL4

AAM74070

HvCOL6

AAL99267

CONSTANS

AT5G15840

AtCOL1

AT5G15850

AtCOL2

AT3G02380

AtCOL3

AT2G24790

AtCOL4

AT5G24930

AtCOL5

AT5G5660

AtCOL6

AT1G25440

AtCOL7

AT1G73870

AtCOL8

AT1G49130

AtCOL9

AT3G07650

AtCOL10

AT5G48250

AtCOL11

AT4G15250

AtCOL12

AT3G21880

AtCOL13

AT2G47890

AtCOL14

AT2G33500

AtCOL15

AT1G28050

AtCOL16

AT1G68520
